# Scientific Items

**Published:** 1880-09-20

**Authors:** 


### SCIENTIFIC ITEMS.

**Why we are Right-Handed.**—Dr. John A. Wyeth, of New York, in an interesting paper on the "Anatomical Reasons for Dextral Preference in Man," which appears in the Annals of the Anatomical and Surgical Society of Brooklyn, N. Y., arrives at the following conclusions:

1. Man is right-handed by preference, as a result of his anatomical development.

2. The arrangement of the embryonic protoplasmic element is such that the liver developing on the right side greatly outgrows its opposing viscus, the spleen, and pushes the heart to the left of its original position in the median line, causing an obliteration of one of the two originally symmetrical arches of the aorta, and an obliquity of the remaining one.

3. This loss of symmetry involves an arrangement of the great vessels of the neck and upper extremities by which the artery carrying blood to the right arm is more favorably situated and receives more blood than the one to the left arm, while the left carotid and vertebral arteries supplying the left half of the encephalon, which presides over motion on the right side of the body, are more favorably situated and convey more blood than the two vessels which have the same distribution on the opposite side.

4. This fact accounts for the development of the left half of the brain in excess of the right.

5. It is not the slight excess in weight of the viscera of the right side of the abdomen, which is given by some to be the cause of right-handedness, who argue from this that man must lean to the left, that is, balance himself upon the left leg, leaving the right extremities freer for action. It is a matter of cubic inches, of bulk, in fact, of cardiac displacement.

6. Education, training by persistent effort, will overcome the natural tendency to dextral preference, and will render the individual more clever with the non-preferred hand, more equally adroit with both sides of his body, more symmetrical in muscular growth; will tend to equalize the two halves of the brain, giving a better cerebral development, and will consequently render him more serviceable to society and himself.—*Journal of Chemistry.*

**Treatment of Stammering.**—Dr. W. B. Hammond, in the British Medical Journal, gives the following practical hints on this subject:

If the attention of the stammerer can be diverted from himself and his articulation, he will often speak to others as calmly and as perfectly as he does to himself when alone. Now there are various ways of accomplishing this object, but the one that I found most effectual was the performance of some slight muscular action synchronously with the articulation of the difficult syllables. The words that trouble me most were those that began with the explosive consonants—those that require the sudden opening of the lips for their enunciation—*b*, *p*, and *t*. I could no more have repeated the alliterative lines, "Peter Piper picked a peck of pickled peppers," etc., to other persons without stammering than I could have walked to the moon, though perfectly able to say the whole piece through without a flaw when speaking alone. With each troublesome word, especially with one beginning a sentence, I made some slight motion with the hand or foot, or even with a single finger, and I found that this plan enabled me to get the word out without stammering. With the enunciation of "Peter," for instance, I would tap the side of my body with the hand just as I opened my lips, and the word was articulated without the least halting. In the procedure, the attention is diverted from the effort to speak to the performance of the muscular action mentioned, and hence the speech becomes more automatic.

No orator thinks of his articulation when he is making a speech; no one in ordinary conversation thinks whether or not he will be able to pronounce a certain word, or to acquit himself well in the management of his tongue and lips. His mind is concerned with his thoughts, with what he is going to say, not with the manner in which he will articulate, and the more thoroughly we can succeed in bringing stammerers into the same way of procedure the more successful shall we be in our efforts to cure them.—*Journal of Chemistry.*

**Photography Under Water.**—Wm. Morris, of Greenock, says the Glasgow News, has made a discovery by which he can photograph underneath the water at a depth of ten fathoms. Two of the negatives he has secured are remarkably distinct, but the others are rather dim, owing to defects in the apparatus, which he hopes to remedy. The camera is enclosed in a water-tight glass case, suspended by the center and enclosed by a cover, that is drawn off after the camera—which is fixed on a loaded tripod—has reached its position.

One of the views taken in the bay shows a sandy bottom, with a number of large boulders covered with seaweed, and an old anchor; and in the shade three mooring cables belonging to small yachts close at hand.

Mr. Morris intends soon to carry out his investigations with improved apparatus when he expects to achieve still greater results.

**Telephone.**—A very curious analogy between the telegraph and the nervous system is found in the telephone, the operation of which is strikingly like that of the human ear.

The transmitter may be called an artificial ear, the metallic disc being the tympanum or drum, the bar magnet taking the place of the bones, and the wire serving as the nerves to transmit the vibrations. Dr. Bell is reported to have said that it was by a patient study of the construction of the human ear that he was led to the invention of this wonderful instrument.—*Ibid.*

**A Winking Portrait.**—A peculiar effect in portraits has been produced by Mr. Simmonar. A negative of the sitter is taken with his eyes open and another with his eyes shut; these are printed on paper in such a way that the front and back images exactly coincide. The two sides of the paper when alternately illuminated present a picture of the same person with his eyes opening and shutting, and if the light is shifted rapidly we have a winking portrait.—*Dental Advertiser.*

